# Supplementary material for: Signatures of adaptation at key insecticide resistance loci in Anopheles gambiae in Southern Ghana revealed by reduced-coverage WGS
Source: Sci Rep. 2024 Apr 15;14:8650. doi: 10.1038/s41598-024-58906-x (PMC11018624; doi:10.1038/s41598-024-58906-x)
Supplement: Supplementary file 2 — Supplementary Figures. [file 41598_2024_58906_MOESM2_ESM.docx]

**
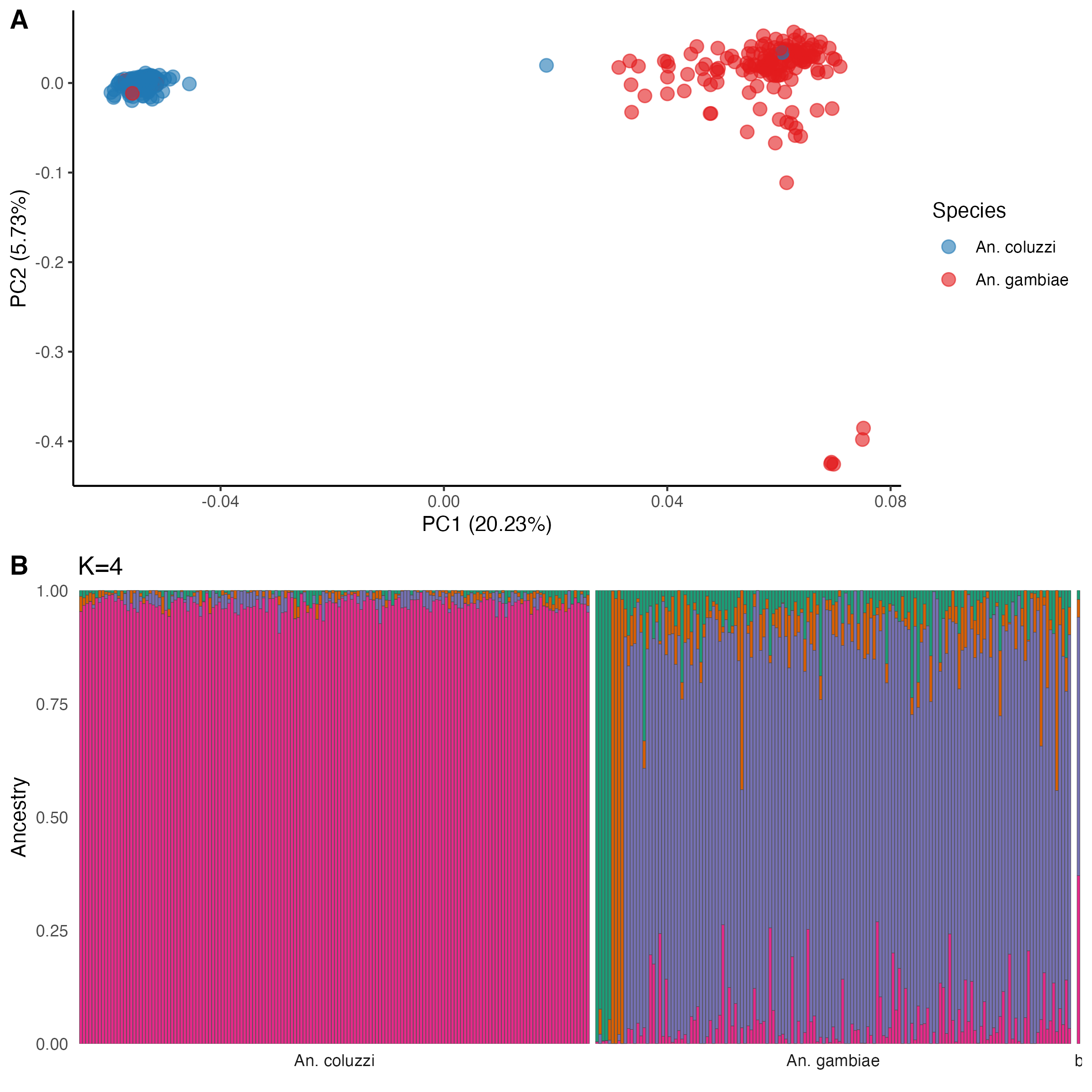
**

**Supplementary Figure 1**: PCA (A) and ADMIXTURE analysis (B) of 314 *An. gambiae* species complex samples. Species colour in (A) denoted by point colour. Cluster colour in (B) denoted by bar colour, windowed by species.

**
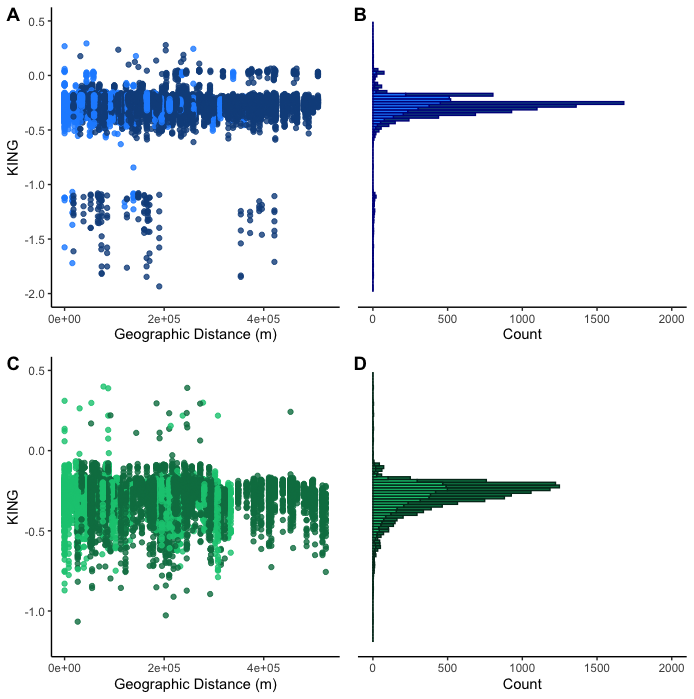
**

**Supplementary Figure 2.**Pairwise KING kinship coefficient measured between pairs of *Anopheles coluzzii* (blue, **A,B**) and *An. gambiae* (green, **C,D**) samples. Panels **A** and **C** denote KING coefficient estimates plotted against geographic distance between samples. Panels **B** and **D** are histograms of KING values. Lighter shading indicates samples from the same ecoregion, darker shading, from different ecoregions.
